# On Why Targets Evoke P3 Components in Prediction Tasks: Drawing an Analogy between Prediction and Matching Tasks

**DOI:** 10.3389/fnhum.2017.00497

**Published:** 2017-10-10

**Authors:** Rolf Verleger, Stephanie Cäsar, Bastian Siller, Kamila Śmigasiewicz

**Affiliations:** ^1^Department of Neurology, University of Lübeck, Lübeck, Germany; ^2^Institute of Psychology II, University of Lübeck, Lübeck, Germany; ^3^Laboratoire de Neuroscience Cognitive, Aix-Marseille Université, CNRS, Marseille, France

**Keywords:** P3, ERPs, prediction, comparison task, matching task, S-R link hypothesis, context updating, P300

## Abstract

P3 is the most conspicuous component in recordings of stimulus-evoked EEG potentials from the human scalp, occurring whenever some task has to be performed with the stimuli. The process underlying P3 has been assumed to be the updating of expectancies. More recently, P3 has been related to decision processing and to activation of established stimulus-response associations (S/R-link hypothesis). However, so far this latter approach has not provided a conception about how to explain the occurrence of P3 with predicted stimuli, although P3 was originally discovered in a prediction task. The present article proposes such a conception. We assume that the internal responses *right* or *wrong* both become associatively linked to each predicted target and that one of these two response alternatives gets activated as a function of match or mismatch of the target to the preceding prediction. This seems similar to comparison tasks where responses depend on the matching of the target stimulus with a preceding first stimulus (S1). Based on this idea, this study compared the effects of frequencies of first events (predictions or S1) on target-evoked P3s in prediction and comparison tasks. Indeed, frequencies not only of targets but also of first events had similar effects across tasks on target-evoked P3s. These results support the notion that P3 evoked by predicted stimuli reflects activation of appropriate internal “match” or “mismatch” responses, which is compatible with S/R-link hypothesis.

## Introduction

The P3 component of event-related potentials (ERPs) was first reported by Sutton et al. (1965) see also Desmedt et al. (1965). Participants had to predict which one of two alternative stimuli would appear. When the sequence was uncertain, stimuli evoked a large positive potential, later termed “P3” (Ritter et al., 1968). Afterwards, another task, the *oddball* task, has become the standard paradigm for eliciting P3. Two stimuli, one frequent, one rare, are presented in random order, requiring different responses (Squires et al., 1975; Duncan-Johnson and Donchin, 1977; Pfefferbaum et al., 1985). Large P3s are evoked by the rare stimuli.

P3 amplitudes may increase at fronto-central sites when stimuli are novel and task-irrelevant, through an increased P3a component, or at centro-parietal sites when stimuli are known and task-relevant, through an increased P3b component (Squires et al., 1975; Gaeta et al., 2003; Dien et al., 2004; Polich, 2007; Sawaki and Katayama, 2009). The mentioned effects in prediction and oddball tasks refer to the P3b component above all. The most influential hypothesis on P3b has proposed that it reflects the updating of expectancies (Donchin, 1981), fitting well the situation of the prediction task. Alternatively, fitting well the oddball task, P3b may reflect processing related to the decision of how to respond (Hillyard and Kutas, 1983; Nieuwenhuis et al., 2005; O’Connell et al., 2012; Twomey et al., 2016). Akin to this notion, we have suggested (entirely independent of our earlier support of a perceptual closure hypothesis, withdrawn in Verleger, 1998; see Sommer et al., 1998) that P3b reflects a short-cut of the decision process, namely reactivation of established, presently inactive stimulus-response (S-R) links (Verleger et al., 2014a, 2016a). To detail, participants will not decide anew in each trial of repetitive tasks about which response is appropriate to which stimulus. Rather, some few fixed S–R links will be established by instruction and practice (Frith and Done, 1986), e.g., *A* → *left*, *B* → *right*. Perceiving a stimulus will call for this link. If not being in an activated state (by having been used in preceding trials) this link will be reactivated, reflected by P3b. There are several other concepts and hypotheses that have been related to P3b, see Verleger and Śmigasiewicz (2016) for a recent review. Here we focus on our S-R link hypothesis because the present research was conducted with the special aim of exploring a possible shortcoming of this hypothesis^1^.

At first sight, the S-R link notion cannot explain why large P3s have been obtained in prediction tasks where targets usually require no responses. For example, in the present study, the two alternative visual target stimuli will have unequal frequencies (80% and 20%), like in some previous studies on the prediction task (Tueting et al., 1970; Verleger et al., 2015; Verleger and Śmigasiewicz, 2016). Let *F* be the frequent target and *I* the infrequent one. Predicting *F* or *I* may be denoted by *f* and *i*. Participants adapted their predictions to these unequal frequencies in those previous studies, making the *f* prediction frequently and the *i* prediction infrequently. Thereby, *f-F* trials occurred often (≈80% guessing probability × 80% target probability = ≈64%), *f-I* rarely (≈16%), as did *i-F* (≈16%), and *i-i* was very rare (≈4%). Target-evoked P3 amplitudes were a function of these frequencies, being large both when targets were infrequent and after infrequent predictions (Tueting et al., 1970; Verleger et al., 2015; Verleger and Śmigasiewicz, 2016). Can this effect be conceived in terms of S-R links? Are there any responses that have to be activated by perceiving rare targets or by perceiving targets after rare predictions? Are the large P3s not simply an indication of surprise reactions, without any response choices involved?

Yet at second sight, there might be responses: targets are supposed to trigger an internal choice whether they do or do not match the prediction and, based on this matching, whether the prediction was right or wrong. This internal choice may be understood as a response. That the response is internal does not seem detrimental, because internal responses like mentally adding “1” have been standard in the oddball task (e.g., Squires et al., 1975; Duncan-Johnson and Donchin, 1977). However, unlike in most studies of the oddball task (see Matt et al., 1992; Rac-Lubashevsky and Kessler, 2016, for exceptions) these right/wrong choices do not only depend on the target but also on a preceding event (which is the participant’s prediction of the target) such that the very same target may evoke two responses, *match* or *mismatch*, depending on what was predicted. Therefore, in terms of S-R link hypothesis, targets will get associative links to these two different responses, and only one of these links will be triggered by the target in any given trial. Thus, rather than focusing on the prediction-target sequence to account for the target-evoked P3 (*f-F*, *f-I*, *i-F*, *i-I*) we focus on the targets and their evoked internal responses (*F*→*match*, *I*→*mismatch*, *F*→*mismatch*, *I*→*match*). These target-response combinations are logically equivalent to the prediction-target sequences and determined by them (e.g., *F*→*match* implies that the prediction was *f*) but describe a process triggered by the target. Indeed, frequency of prediction becomes frequency of responding under this view: *match* and *mismatch* are frequent and infrequent responses with the frequent target *F* but infrequent and frequent responses with the infrequent target *I*. Thus, what is relevant is the conditional response frequency given the target. This allows for a description of effects on P3 amplitudes in terms of S-R links: because *F*→*match* is frequent and *F*→*mismatch* is rare, the *F*→*mismatch* S-R link needs more reactivation when occurring and therefore, evokes a larger P3b than *F*→*match*. Likewise, being only rarely activated, any links from the rare target *I* need reactivating, both to *mismatch* and to *match*, reflected by large P3bs.

**Figure 1:**
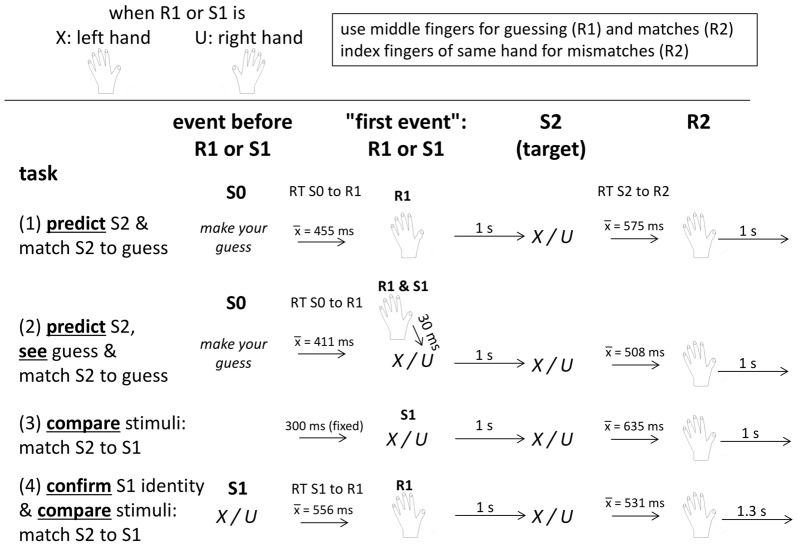
Schematic description of trial structure and timing in each of the four task versions. See text for details. “S0” means the stimulus before R1 or S1. “S1” (stimulus 1) is a blue X or U, “R1” (response 1) is the prediction (in the upper two tasks) or the response to S1 (in the bottom task), “S2” (stimulus 2) is a black X or U, and “R2” (response 2) is the response to S2 indicating whether S2 matches the first event(s) R1 (tasks 1, 2, 4) or S1 (tasks 2, 3, 4).

If indeed reflecting activation of match or mismatch responses, P3b evoked by targets to be matched to a preceding predictive key-press should be the same, and obey to the same regularities, as P3b evoked by targets to be matched to a preceding stimulus. Demonstrating this similarity between prediction and comparison tasks is the major aim of the present study. To conduct a proper comparison between tasks, the prediction and comparison tasks must be made as similar as possible to each other in terms of timing and response requirements, as shown in Figure 1, the major difference being that the event that precedes the target (S2) is a predictive key-press (R1) in the prediction task but a first stimulus (S1) in the comparison task. Target frequency was equally varied in the two tasks by presenting one of the two targets S2 frequently and the other rarely. Similarly, conditional frequency of *match* vs. *mismatch* responses to a given target was varied by having participants make frequent and rare predictions (R1) in the prediction task and by presenting one of the two stimuli (S1) frequently and the other rarely in the comparison task. We expect to replicate the finding of our preceding studies (Verleger et al., 2015; Verleger and Śmigasiewicz, 2016) that S2-evoked P3b is not only (trivially) larger with rare than frequent targets but also with rare than frequent responses to a given target (conceived as rare vs. frequent predictions in those previous studies). That is, we expect to replicate that the type of response (*match* vs. *mismatch*) should not matter *per se*, i.e., it should not matter whether a target was or was not predicted. Rather the frequency of the target-response combination should be decisive. New in the present study is that we compare prediction and comparison tasks. We expect that effects on S2-evoked P3 of target (S2) frequencies and of conditional response frequencies with S2 (induced by R1 or S1) will not differ between prediction and comparison tasks. Moreover, P3b amplitudes should be of the same size in the two tasks.

Effects on response times (RTs) and error rates in response to target stimuli were expected to parallel the P3 results. Making responses to predicted targets (R2, see Figure 1) has not often been required (see Verleger and Cohen, 1978, for an exception). A previous study, comparing P3 in prediction tasks with and without R2 responses (Verleger and Śmigasiewicz, 2016) found enhanced S2-evoked P3 amplitudes when responses were required, yet this enhancement did not interact with the effects of stimulus frequency and correctness of guess. Here, we aimed at adapting response requirements closely to the task requirement of keeping the first events (S1 or R1) in working memory. As R1 in the prediction task, participants pressed a key with the left hand to predict an *X* and a key with the right hand to predict a *U*. To embody the working-memory process, responses (R2) to the target were then made with the hand used for prediction: if prediction was correct, the same key had to be pressed again (with the middle finger, also used for making the prediction) and if the prediction turned out to be wrong the neighboring key had to be pressed (with the index finger). Likewise, in the comparison task, the hand to be used for responding to the target S2 was determined by the first event: if S1 was an *X*, responses to the following target had to be made with the left hand, and if S1 was a *U*, responses to the target had to be made with the right hand (match: middle finger; mismatch: index finger). We expected that RTs and error rates would be equally affected across the two tasks by target frequency and by conditional response frequency to a given target.

## Materials and Methods

### Participants

Sixteen university students participated (eight females and eight males, aged 23 years on average, SD = 2 years). One other participant had to be replaced because of equipment failure during recording. Twelve were right-handed (scores of 88 ± 13 in the Edinburgh Inventory, Oldfield, 1971), four were left-handed (−73 ± 13). All participants reported normal or corrected-to-normal vision and no history of neurological disorders. The experiment was conducted according to the Helsinki Declaration. The general procedure in our EEG lab was approved by the Ethic Committee of the University of Lübeck (file # 05-043). Written information, approved by the Ethic Committee, was presented to each participant and was signed as statement of informed consent. Participants were paid depending on their success in guessing in the prediction task in order to keep participants interested in guessing and in outcome of their guesses, and to prevent them from mechanically pressing some key for prediction. To keep payment structure identical between tasks, participants were likewise paid for the occurrence of objective matches between first and second stimulus in the comparison task. Participants earned 15.91 € on average (± 0.65 €).

### Stimuli and Procedure

The experiment consisted of four blocks, namely of two versions each of the prediction and comparison tasks. Each of the four blocks lasted for about 15 min. Participants were seated in a comfortable armchair in a darkened room, with about 1.1 m viewing distance from the 17″ computer screen, and held a computer ISO QWERTZ keyboard on their lap. They were informed by written instruction on the screen that the experiment aimed at comparing EEG potentials between two different tasks, prediction and matching, that stimuli were two letters (*X* and *U*), one being frequent and the other rare (80%/20%), alternating between participants, that the left hand was assigned to *X* and the right hand to *U*, and that the middle fingers were to be used for indicating predictions and matches, and the index fingers for indicating mismatches. The ↓ and ← navigation keys were used for right-hand responses, and the left *shift* and < keys (which are next to each other on ISO QWERTZ keyboards) for left-hand responses. In any trial, only one hand had to be used, as defined by the first event (prediction or S1). Then the four tasks were presented, as outlined in Figure 1. Each task consisted of 250 trials. The *X* or *U* letters were always displayed at screen center in Helvetica, 35 points, for 200 ms on the light gray screen.

To compare the prediction and comparison tasks, we made them as similar as possible (see Figure 1): presenting the two targets with 80% vs. 20% frequency in both tasks, rewarding correct guesses and matches in both tasks, trying to equalize the intertrial interval across both tasks, and requiring match/mismatch responses to targets in both tasks. To further increase similarity of the two tasks, either task was presented in a second version. Whereas the pure versions included either a key-press (R1) or a stimulus (S1) before the targets, the second versions included both R1 and S1 (although necessarily in different functions in the two tasks, see below).

Before the prediction tasks, participants were informed that the task was a gamble, that they had to guess by key-press which of the two letters *X* or *U* would occur, that accurate guesses would yield 2 cents for the frequent letter and 8 cents for the rare one, and no money would be lost after inaccurate guesses. Because of this payoff scheme, predicting the rare letter was as rational as predicting the frequent one: predicting the frequent stimulus in five of five trials would yield 8 cents (being probably presented in four out of five trials, due to its 80% probability; 4 × 2 cents = 8 cents) and predicting the rare stimulus in five of five trials would also yield 8 cents (being probably presented in one out of five trials, due to its 20% probability; 1 × 8 cents = 8 cents). Thus, participants could adapt their prediction behavior to the objective probabilities without risk of losing money. After every 20 trials and at task ending, summary feedback was given, separately for frequent and rare outcomes, on the number of correct guesses and on the amount of money earned. Each trial in the prediction tasks started with a guess prompt below screen center (“S0” in Figure 1; “guess, please” in German, in black 20 pt. font) displaying the two letters left and right below the prompt, as a reminder about which hand was assigned to which letter. To prevent premature mechanistic guessing, error messages (“pressed too early”, in German) appeared in large red 30 pt. font for 4 s whenever the keys were pressed before onset of the guess prompt. Key-pressing with the left or right middle finger (indicating prediction of an *X* or a *U*) blanked the screen and was followed in the pure Prediction task (*Predict*) after 1 s by a black *X* or *U* as target stimulus. Guess correctness had to be indicated by key-press after the target, using the same middle finger that made the prediction if the prediction was correct, or the neighboring index finger if the prediction was wrong. The program waited until the correct key was pressed. The guess prompt of the next trial was presented 1 s later. The *Predict and See* task was identical to the pure prediction task, except for presenting an S1, like in the comparison tasks: prompted by the prediction key-press, a blue *X* or *U* (mirroring the prediction) was presented 30 ms afterwards, followed 1 s after its onset by the target.

Before the comparison tasks, participants were informed that two stimuli would be presented in a row, that they had to press a key with the middle or index finger after the second stimulus according to whether S2 did or did not match S1, and that the hand used for key-pressing was defined by S1 (*X* left, *U* right). To keep payoff similar to the prediction task, matches of S1 and S2 stimuli yielded gains, 2 cents. with frequent stimuli and 8 cents. with infrequent ones, and mismatching S1 and S2 yielded 0 cents., with summary feedback again provided after every 20 trials and at task ending. Trials started with a blue *X* or *U* as S1. In the pure *Compare* task, the black target letter followed 1 s after onset of the first letter, and any key-press before onset of the target letter prompted the “pressed too early” message. In *Confirm and Compare* (confirm S1 identity and match S2 to S1), S1 had to be responded by pressing the corresponding *X* or *U* key (with the middle finger) and the target letter followed 1 s after the correct key was pressed. In both comparison tasks, the *match* (middle finger) or *no-match* (index finger) keys had to be pressed after the second letter with the hand defined by S1. The next trial started 1.3 s after a correct key-press, somewhat more than the 1 s interval in the prediction tasks, in order to compensate for the time needed for making the guess in response to the guess prompt^2^.

It was balanced across participants whether *X* or *U* was frequent (thereby, whether the left or right hand was frequently used) whether prediction or matching tasks came first, and whether, within these parts, the S1-*or*-R1 tasks (pure *Prediction* and pure* Comparison*) or the S1-*and*-R1 tasks (*Predict and See* and *Confirm and Compare*) came first. Presentation^®^ software 17.2^3^ was used to present the stimuli, register responses, and send stimulus and response codes to another computer where codes were stored with the recorded EEG. The letters to be presented as target and as S1 in the comparison tasks were randomly chosen in each trial independently of each other with 80/20 probabilities. Thereby, the expected probability of S1/R1 and the target matching was 64% + 4*%* = 68%, and the expected payoff for a trial was 64% × 2 cents + 4% × 8 cents = 0.128 + 0.032 cents = 0.16 cents. Thus, the expected value for the 1000 trials of the session (4 tasks × 250 trials) was 16 €, which comes close to what participants actually earned (see above).

### Analysis of Behavior and of Event-Combination Frequencies

There were four event combinations which may be written as *f*→*F*, *i*→*F*, *f*→*I*, *i*→*I* (*f* = frequent, *i* = infrequent, denoting the first event, prediction or S1, with lower-case letters and the S2 targets with upper-case letters) leading to the four target-response combinations *F*→*match*, *F*→*mismatch*, *I*→*mismatch*, *I*→*match*. Frequencies of these combinations were determined by the computer program in the comparison tasks but depended on participants’ guessing behavior in the prediction tasks and were, therefore, assessed for each participant. Separately for each of these four event combinations in each task, RTs to S2 were averaged across correctly responded trials. Percentages of incorrectly responded trials were determined, separately for using the wrong finger of the correct hand and for other errors. RTs to the guess prompt (in the prediction tasks) and to correctly responded S1 (in the *Confirm and Compare* task) were likewise averaged across trials.

### EEG Recording and Analysis

EEG was recorded with Ag/AgCl electrodes (Easycap)^4^ from 60 scalp sites, including eight midline positions from AFz to Oz and 26 pairs of symmetric left and right sites. Additional electrodes were placed at the nose-tip for off-line reference and at Fpz as connection to ground. On-line reference was Fz. For artifact control, EOG was recorded, vertically (vEOG) from above vs. below the right eye and horizontally (hEOG) from positions next to the left and right tails of the eyes. Data were amplified from DC to 250 Hz by a BrainAmp MR plus and stored at 500 Hz per channel. Off-line processing was done with Brain-Vision Analyzer software (version 2.03). Data were re-referenced to the nose-tip, low-pass filtered at 25 Hz, and segmented to epochs of 2.1 s duration, from 1100 ms before S2 onset to 1 s afterwards. These epochs were edited for artifacts. First, epochs were rejected as gross artifacts when consecutive data points differed by more than 50 μV (except EOG, lest trials would be rejected for blinks). Then, ocular artifacts were corrected by using the linear regression method implemented in the BrainAnalyzer software. Finally, data were referred to the mean amplitude of the first 100 ms before S2 onset as baseline in each channel, and trials were rejected when voltages exceeded ± 150 μV in any EEG channel.

EEG data were then averaged over those trials where targets were correctly responded, separately for the four event combinations in the four tasks. Parameters were measured in these averaged waveforms. Mean numbers of trials included in averages were, across the four tasks and participants, 140, 42, 29, and 11 for *F*→*match*, *F*→*mismatch*, *I*→*mismatch*, *I*→*match*. Minimum numbers included were 100, 16, 5, and 2. Because of these low numbers (even though in single cases only), leading to appreciable noise in the signal, P3 amplitudes were measured in a broad window (chosen after inspection of the grand means), as mean amplitudes 300–500 ms after target onset averaged across five recording sites, which were CPz and its four nearest neighbors Cz, Pz, CP1, CP2. CPz was chosen as midpoint because P3b amplitudes had been largest at this site in our previous studies (e.g., Verleger and Śmigasiewicz, 2016) and indeed were largest there in the present data as well.

### Statistical Analysis

Event-combination frequencies, RTs to targets, arc-sinus transformed error rates and P3 amplitudes were evaluated by analysis of variance (ANOVAs; IBM SPSS Statistics 22) with the factors Task (prediction vs. comparison), Task Version (one vs. two first events, i.e., S1 *or* R1 in pure *Prediction* and *Comparison* vs. S1 *and* R1 in *Predict and See* and *Confirm and Compare*), S2 Frequency (frequent vs. rare) and Conditional Response Frequency (to a given S2: frequent vs. rare). To clarify interactions, ANOVAs were conducted on the single levels of interacting factors.

Besides, RTs to the guess prompt were evaluated by ANOVA with the two factors Task Version (*Predict* vs. *Predict and See*) and R1 Frequency (frequent vs. rare). Then, RTs to the guess prompt were averaged across these two tasks and compared with RTs to S1 in *Confirm and Compare* by ANOVA with the two factors Task (prediction vs. comparison) and R1/S1 Frequency (frequent vs. rare).

## Results

### Behavior

#### Event-Combination Frequencies

**Figure 2:**
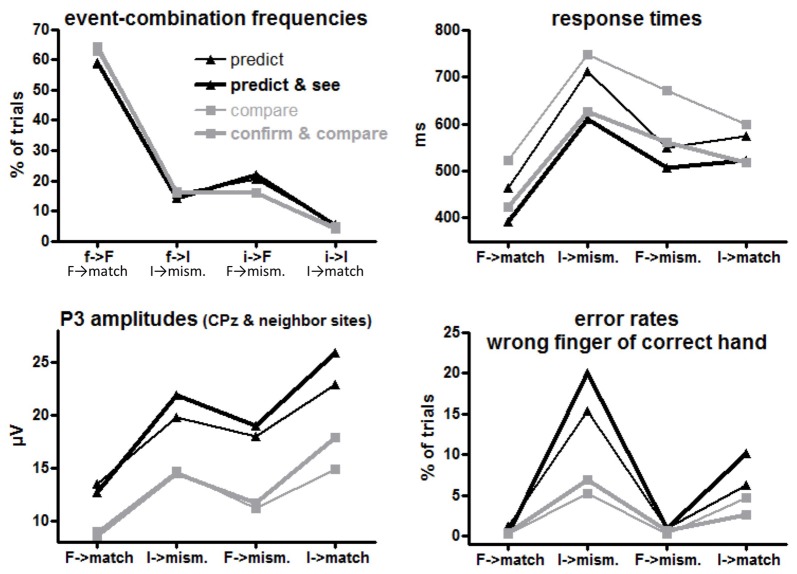
Mean event frequencies, response times (RTs), error rates and P3 amplitudes for each first event/target combination or the equivalent target-response combination in each task version. *F* and *I* on *x* axis denote frequent and infrequent targets, *f* and *i* denote frequent and infrequent first events (prediction or S1), *match* and *mismatch* denote the corresponding response requirements to the targets. Black and gray lines denote values from prediction and comparison tasks, respectively. Thin and bold lines denote values from versions with one or two first events (R1 and S1), respectively.

*f*→*F*, *f*→*I*, *i*→*F*, *i*→*I* (= *F*→*match*, *I*→*mismatch*, *F*→*mismatch*, *I*→*match* response requirements) occurred in 64%, 16%, 16% and 4% of trials in the comparison tasks (where S1 and S2 were computer-determined) and in 59%, 15%, 21%, and 5% of trials in the prediction tasks (where only S2 was computer-determined while R1 was chosen by participants), see upper left panel of Figure 2. By design, the frequencies of the four combinations differed, *F*_(1,15)_ ≥ 385, *p* < 0.001 for main effects of R1/S1 Frequency (= Conditional Response Frequency) and S2 Frequency and their interaction. Of more interest, participants made *frequent* predictions less and, correspondingly, *rare* predictions more often than their actual probabilities (Task × R1/S1 Frequency *F*_(1,15)_ = 7.4, *p* = 0.02). In absolute numbers, this effect was larger for frequent than rare events, resulting in a triple interaction of Task × R1/S1 Frequency × S2 Frequency, *F*_(1,15)_ = 5.9, *p* = 0.03. Effects of Task Version did not reach significance, *F*_(1,15)_ ≤ 3.7, *p* ≥ 0.07.

#### Response Times

Mean RTs to the target S2 are displayed in the upper right panel of Figure 2 and ANOVA results are compiled in Table 1.

**Table 1:** ANOVA results on response times (RTs) to S2.

	Main effects of row factors	Task	Task version	Task × Task version
Main effects of column factors		2.4	**13.6**	0.7
			**0.002**	
Condit. Response Frequency	0.0	1.0	2.7	0.4
S2 Frequency	**54.5**	**6.7**	0.3	0.4
	**<0.001**	**0.02**		
Cond. Resp. Freq. × S2 Frequency	**58.5**	1.1	0.6	0.3
	**<0.001**			

*Note. ANOVA on RTs to the target (S2; F and p values). Effects involving Task, Task Version, and their interaction are entered in columns, and effects involving Conditional Response Frequency, S2 Frequency, and their interaction are entered in rows. F-values and p-values are printed in bold when p ≤ 0.050. p-values were entered when p ≤ 10. Degrees of freedom were 1/15 throughout*.

The large effect of S2 Frequency, *F*_(1,15)_ = 54.5, *p* < 0.001, was qualified by its interaction with Conditional Response Frequency, *F*_(1,15)_ = 58.5, *p* < 0.001: responses were much faster when S2 was frequent than when S2 was rare for conditionally frequent responses (*F*→*match* vs. *I*→*mismatch*; simple effect of S2 Frequency *F*_(1,15)_ = 107.9, *p* < 0.001). For conditionally infrequent responses (*F*→*mismatch* and *I*→*match*) RTs lay in-between these two extremes (both slower than *F*→*match* and faster than *I*→*mismatch* in pair-wise comparisons, *F*_(1,15)_ ≥ 19.8, *p* < 0.001) and did not differ from each other (effect of S2 Frequency: *F*_(1,15)_ = 0.8, n.s.) except for the interaction with Task described next.

The tasks differed in one respect: with frequent targets, responses were somewhat faster in prediction than comparison tasks, S2 Frequency × Task *F*_(1,15)_ = 6.7, *p* = 0.02; simple effect of Task for frequent S2 *F*_(1,15)_ = 4.9, *p* = 0.04; for rare S2 *F*_(1,15)_ = 0.4, n.s. Although the S2 Frequency × Task effect did not differ in extent for frequent and rare responses (*F*_(1,15)_ = 1.1, n.s., for the triple interaction), this effect is less clearly seen in Figure 2 for frequent responses: the large S2 Frequency effect (*F*→*match* faster than *I*→*mismatch*) only was somewhat larger with prediction tasks (*F*_(1,15)_ = 73.8, *p* < 0.001) than with comparison tasks (*F*_(1,15)_ = 45.2, *p* < 0.001). The effect is more distinctly seen for rare responses *F*→*mismatch* and *I*→*match*, where RTs were equal for frequent and rare S2 in prediction tasks (*F*_(1,15)_ = 0.7, n.s.) but were slower with frequent S2 than rare S2 in comparison tasks (*F*_(1,15)_ = 5.9, *p* = 0.03).

Finally, responses were overall faster in those task versions where both S1 and R1 were present than when either S1 or R1 was present, *F*_(1,15)_ = 13.6, *p* = 0.002 (bold vs. thin lines in Figure 2). This effect did not differ between prediction and comparison tasks (*F*_(1,15)_ ≤ 0.7, n.s., in rightmost column of Table 1).

#### Error Rates

Errors in responding to the target S2 might be committed by choosing the wrong finger of the correct hand (as predetermined by the first event) or by using the other hand. Most errors were of the first type, occurring on average in 5.5% of trials. Other-hand errors occurred in 0.5% of trials only, and will not be detailed. Mean rates of wrong-finger errors are displayed in the lower right panel of Figure 2 and ANOVA results on arcsine-transformed values are compiled in Table 2.

**Table 2:** ANOVA results on arcsine-transformed percentages of wrong-finger responses to S2.

	Main effects of row factors	Task	Task version	Task × Task version
Main effects of column factors		**7.2**	2.4	4.2
		**0.02**		0.06
Condit. Response Frequency	**9.6**	3.2	0.3	0.3
	**0.007**	0.09		
S2 Frequency	**28.6**	**6.0**	3.0	**5.7**
	**<0.001**	**0.03**		**0.03**
Cond. Resp. Freq. × S2 Frequency	**10.9**	3.5	0.9	0.2
	**0.005**	0.08		

*Note. ANOVA on arcsine-transformed error rates to the target (S2) in responding with the wrong finger of the correct hand (F and p values). (Other errors were negligible). Effects involving Task, Task Version, and their interaction are entered in columns, and effects involving Conditional Response Frequency, S2 Frequency, and their interaction are entered in rows. F-values and p-values are printed in bold when p ≤ 0.050. p-values were entered when p ≤ 10. Degrees of freedom were 1/15 throughout*.

More errors were committed with rare than frequent targets (S2 Frequency *F*_(1,15)_ = 28.6, *p* < 0.001). This effect was larger with frequent than with rare responses (Conditional Response Frequency × S2 Frequency, *F*_(1,15)_ = 10.9, *p* = 0.005; simple effect of S2 Frequency with frequent responses *F*_(1,15)_ = 46.0, *p* < 0.001; rare responses *F*_(1,15)_ = 7.6, *p* = 0.02). This was also reflected in a main effect of Conditional Response Frequency, *F*_(1,15)_ = 9.6, *p* = 0.007, but as reflected by its interaction with S2 Frequency, Conditional Response Frequency, unlike with RTs, did not have effects when S2 was frequent (simple effect: *F*_(1,15)_ = 0.4, n.s.) where error rates were close to zero throughout.

Error rates were higher in prediction than comparison tasks, *F*_(1,15)_ = 7.2, *p* = 0.02. This effect was obviously (Figure 2) larger with rare than with frequent S2 (Task × S2 Frequency *F*_(1,15)_ = 6.0, *p* = 0.03). Yet, even though error rates were close to zero with frequent S2, the task effect was clearly significant, too: frequent S2 *F*_(1,15)_ = 11.0, *p* = 0.005, rare S2 *F*_(1,15)_ = 6.6, *p* = 0.02.

Finally, error rates differed between versions of the prediction task: more errors were made with rare S2 in the *Predict and See* task than in the *Predict* task (black bold line higher than black thin line in Figure 2) while there was no corresponding effect of Task Version in the comparison tasks (Task × Task Version × S2 Frequency *F*_(1,15)_ = 5.7, *p* = 0.03; Task Version × S2 Frequency in prediction tasks *F*_(1,15)_ = 5.5, *p* = 0.03; in comparison tasks *F*_(1,15)_ = 0.1, n.s.).

#### RTs to Guess Prompts and S1

Mean RTs to guess prompts (values inserted in Figure 1) were 455 ms in *Predict* and 411 ms in *Predict and See*. There was no reliable difference between these two task versions nor between frequent and rare guesses (all *F*_(1,15)_ ≤ 1.4, n.s.). Mean RTs to S1 in *Confirm and Compare* were 556 ms (also inserted in Figure 1). This was longer than the average RTs to guess prompts, both for frequent key-presses (by 91 ms) and even more for rare ones (by 251 ms), leading to significant effects in the common ANOVA for main effects both of Task and of R1/S1 Frequency, and for their interaction (all *F*_(1,15)_ values ≥ 13.2, *p* ≤ 0.002).

### ERPs

#### S2-Evoked P3 Amplitudes

**Figure 3:**
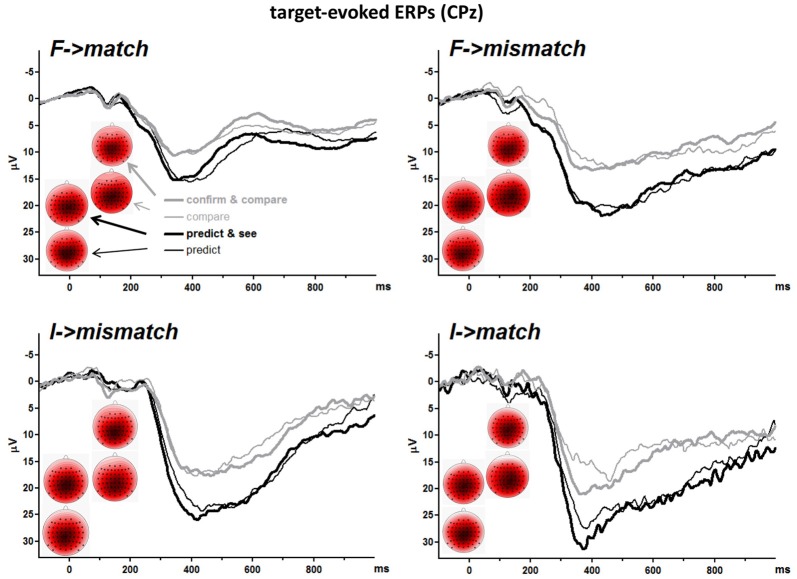
Grand averages of target-evoked event-related potentials (ERPs) recorded from CPz. Each panel depicts one target-response combination, e.g., *F*→*match* displays ERPs evoked by frequent targets that matched the first event. *F* and *I* denote frequent and infrequent targets. Like in Figure 2, black and gray lines denote values from prediction and comparison tasks, and thin and bold lines denote values from versions with one or two first events (R1 and S1). Unit of x-axis is ms, with the zero point denoting target (S2) onset. Unit of *y-axis* is μV, with negative polarity plotted upwards. The scalp maps display topographic distributions of mean amplitudes 300–500 ms after target onsets. View is from above, with Cz in the center and ear level (=120°) at the outer rim, nose is above. Colors are individually scaled in each map to range symmetrically between ± 125% of its absolute maximum amplitude (e.g., with minimum and maximum at −8 μV and +4.5 μV, the range would be ± 10 μV; 125% was chosen rather than 100% to avoid oversaturated coloring). Blue is negative, white is zero, red is positive.

Grand means of S2-evoked waveforms recorded from CPz are displayed in Figure 3. As shown by the maps (Figure 3) P3 amplitudes were indeed largest at CPz. Mean amplitudes were averaged from CPz and its four nearest neighbors Cz, Pz, CP1, CP2 to reduce any noise. These amplitudes are displayed in the lower left panel of Figure 2 and ANOVA results are compiled in Table 3. S2 Frequency and Conditional Response Frequency had significant effects on these S2-evoked amplitudes but, unlike with RTs and error rates, these effects did not interact (*F* ≤ 0.9 in bottom row of Table 3). Thus, S2-evoked P3 amplitudes were larger with rare than frequent S2, *F*_(1,15)_ = 85.3, *p* < 0.001, equally with frequent and rare responses, and were larger with conditionally rare than frequent responses, *F*_(1,15)_ = 15.2, *p* = 0.001, equally with frequent and rare S2. Moreover, P3 amplitudes were larger in prediction than comparison tasks, *F*_(1,15)_ = 31.7, *p* < 0.001. As reflected by the absence of interactions with the Task factor, *F*_(1,15)_ ≤ 4.1, *p* ≥ 0.06, this was equally true for all four combinations of S2 frequency and conditional response frequency. There was no reliable difference between the two task versions, *F*_(1,15)_ ≤ 4.0, *p* ≥ 0.07. These latter non-significant tendencies reflected a possibly larger effect of S2 Frequency with prediction than comparison tasks, and possibly larger effects of both S2 Frequency and Conditional Response Frequency with the S1&R1 versions than with the single-first-event versions of both tasks.

**Table 3:** ANOVA results on target-evoked P3 amplitudes (averaged across CPz, Cz, Pz, CP1, Cp2).

	Main effects of row factors	Task	Task version	Task × Task version
Main effects of column factors		**31.7**	2.9	0.2
		**<0.001**		
Condit. Response Frequency	**15.2**	2.6	3.5	0.1
	**0.001**		0.08	
S2 Frequency	**85.3**	4.1	4.0	0.4
	**<0.001**	0.06	0.07	
Cond. Resp. Freq. × S2 Frequency	0.7	0.9	0.3	0.6

*Note. ANOVA on target-evoked P3 amplitudes (F and p values). Effects involving Task, Task Version, and their interaction are entered in columns, and effects involving Conditional Response Frequency, S2 Frequency, and their interaction are entered in rows. F-values and p-values are printed in bold when p ≤ 0.050. p-values were entered when p ≤ 10. Degrees of freedom were 1/15 throughout*.

#### ERPs before S2

**Figure 4:**
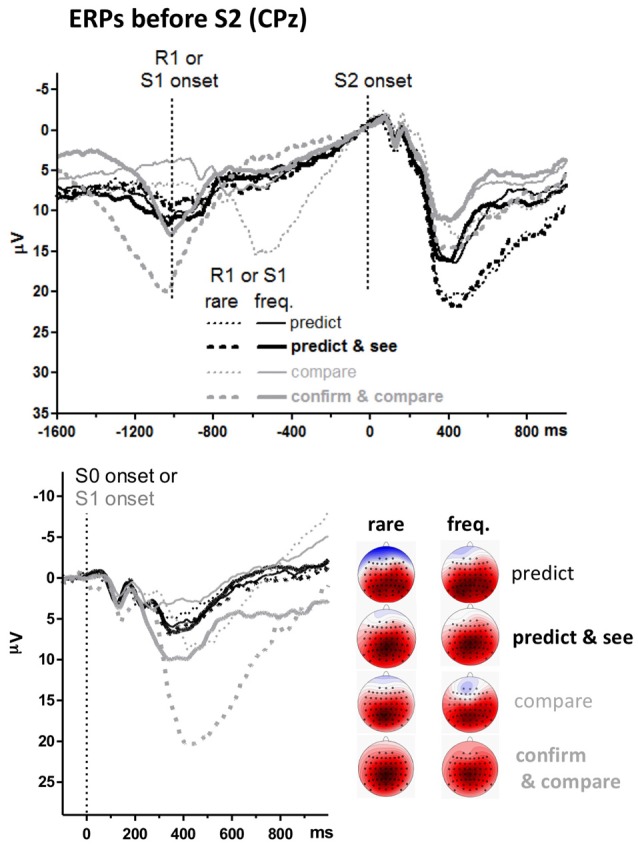
Grand averages of ERPs before targets, recorded from CPz. Like in Figures 2, 3, black and gray lines represent prediction and comparison tasks, and thin and bold lines represent versions with one or two first events (R1 and S1). Additionally, solid and dashed lines denote values from frequent and rare first events. Unit of *y*-axis is μV, with negative polarity plotted upwards. Unit of *x-axis* is ms. Upper panel: time-point zero is target (S2) onset. Different from Figure 3, the epoch before target onset is displayed, and values are pooled across frequent and rare targets. Lower panel: time-point zero is the first stimulus in the trial (S0 in prediction tasks, S1 in comparison tasks). The scalp maps display topographic distributions of mean amplitudes 300–500 ms after first-stimulus onsets. See legend of Figure 3 for details on map scaling.

The unexpected main effect of Task on S2-evoked P3 might be related to differences in ERPs preceding S2. The upper panel of Figure 4 shows ERPs 1.6 s before S2 onsets. Data were pooled from frequent and rare S2 (i.e., from upper and lower panels of the S2-evoked data shown in Figure 3, separately for left and right side of that figure) because participants could not know the target before its onset. The event at −1000 ms is S1 for the pure* compare* task and R1 in the other three tasks (see Figure 1). There is a slow negativity in all displayed waveforms, starting at about 500 ms before S2 onset and culminating at S2 onset, reflecting expectancy of S2 and possibly preparation for the R2 response. This is a CNV (contingent negative variation, Walter et al., 1964) like in in our previous study on the prediction task (Verleger et al., 2015). Since CNV reaches its peak at the 100 ms before S2 onset which epoch is here used as baseline, voltages start in the positive range and align at zero voltage at S2 onset. CNV amplitudes are actually difficult to measure because it appears almost impossible to find a baseline common to all conditions, mainly because of differences in S0- or S1-evoked positive potentials (see below). But it appears that there are no consistent differences in CNV that might explain why the following S2-evoked P3 amplitudes were constantly larger in the prediction than the comparison tasks.

Positive potentials are visible before the CNV. One such potential reaches its peak at −600 ms with rare S1 in the pure* compare* task. This is obviously the P3 evoked by rare S1s presented at −1000 ms. Similarly, there is a distinct positive potential at −1000 ms with rare S1 in the *confirm and compare* task. This is obviously the P3 evoked by these rare S1s presented at about −1500 ms, because −1000 ms in this task is R1, with S1 being about 500 ms earlier (see Figure 1). Nothing of this kind is seen in the *predict and see* task: even though an S1 is presented in this task, 30 ms after R1, rare R1 and S1 do not evoke any P3-like positivity.

To analyze these stimulus-evoked positivities before the target stimuli, ERPs evoked by the first stimuli in each trial are overlaid in the lower panel. These first stimuli are S0 in the prediction tasks (“guess, please”) and S1 (blue *X* or *U*) in the comparison tasks. Thus, these ERPs are identical to the epoch from −1100 ms to 0 ms of the upper panel for the *compare* task where S1 is precisely 1000 ms before S2 onset (except for the difference in baseline). Data of the other three tasks are time-locked to S0 or S1 which occurred about 1500 ms before S2 onsets (see Figure 1) which data are time-jittered with respect to that onset in the upper panel. P3s evoked by these first S0 or S1 were measured like the target S2 stimuli, 300–500 ms after stimulus onset at CPz and its four neighbors Cz, Pz, CP1, CP2 (see the maps in Figure 4, showing largest amplitudes at CPz), and entered to a three-way ANOVA with the factors Task and Version, as above, and Probability (frequent, rare). The Probability factor meant response probability with the S0 stimuli in the prediction tasks, stimulus probability with S1 in the *compare* task, and stimulus and response probability with S1 in the *confirm and compare* task. All three main effects (*F*_(1,15)_ ≥ 18.1, *p* ≤ 0.001), all three second-order interactions (*F*_(1,15)_ ≥ 18.6, *p* ≤ 0.001) and the third-order interaction (*F*_(1,15)_ = 5.1, *p* = 0.04) were significant. Separate ANOVAs for the two tasks showed that no effects were significant in the prediction tasks (*F*_(1,15)_ ≤ 2.8, *p* ≥ 0.12 for effects of Version and Probability in common analysis of the two task versions) whereas both the main effects of Version and Probability and their interaction were significant in the matching tasks, *F*_(1,15)_ ≥ 17.0, *p* ≤ 0.001, indicating that S1-evoked P3s were larger with rare than frequent S1 in the matching tasks, and more so in *confirm and compare* than in pure* compare*.

## Discussion

P3 evoked by target events that had to be predicted was conceived in this study as reflecting the activation of an internal response (R2) indicating the matching or mismatching of the target (S2) to the preceding prediction (R1). To test this proposal, this prediction task, with its putative matching of S2 to R1, was brought into a common experimental set-up with an explicit comparison task where the target S2 had to be compared with a first stimulus S1 (matching of S2 to S1). Thus, the responses required to the target S2 as a function of a first event were *match* and *mismatch* responses in both tasks while the type of first event differed between tasks (prediction key-press R1 vs. stimulus S1). In order to further increase similarity between the two tasks, second versions were constructed, with the second version of the prediction task also presenting an S1 and the second version of the comparison task also requiring an R1.

We had expected that S2(target)-evoked P3s would be affected by S2 frequency and by conditional response frequency of *match* vs. *mismatch* to similar extents in the prediction task as in the comparison task. This expectation was borne out. Further we had expected that S2-evoked P3 amplitudes were equally large in prediction and comparison tasks. This expectation was disconfirmed, with S2-evoked P3 amplitudes being throughout larger in prediction than comparison tasks.

### Similar Effects of Event Frequencies in the Two Tasks

The effects of target frequency and of conditional response frequency (*match* and *mismatch* as governed by prediction frequency) on target-evoked P3 in the prediction task principally replicate results reported by Tueting et al. (1970) and more recently by Verleger et al. (2015) and Verleger and Śmigasiewicz (2016). What we here interpret as effects of conditional response frequency was interpreted as effects of prediction frequency in those articles. The present study applied these manipulations of frequency to a comparison task where the imperative S2 was preceded by an S1. Effects of S2 frequency and conditional response frequency on S2-evoked P3 were highly similar between tasks, confirming the idea that these effects are caused by the same mechanisms. This result opens the way for interpreting the prediction-task results in terms of our S-R link hypothesis on P3 (Verleger et al., 2014a,b, 2016a), positing the reactivation of target-response links as the mechanism underlying the occurrence of P3 with predicted stimuli.

It may be suggested that these effects of conditional response frequencies are simply effects of preceding stimulus sequences, as studied since decades in oddball tasks. To detail, stimulus alternations have often been found to evoke larger P3s than stimulus repetitions (e.g., Squires et al., 1976; Verleger, 1991; Sommer et al., 1999; Kolossa et al., 2013). Therefore, P3 amplitudes evoked by the frequent target *F* are expected to be larger with *i*→*F* (*F*→*mismatch*) than with *f*→*F* (*F*→*match*) which was the case, and P3 amplitudes evoked by the infrequent target *I* are expected to be larger with *f*→*I* (*I*→*mismatch*) than with *i*→*I* (*I*→*match*) which was not the case but may have been obscured by an overlapping opposite effect of probability, enhancing P3 with the extremely rare *i*→*I*. If indeed due to such stimulus sequence effects, the present results would not provide evidence in favor or against S-R link hypothesis. But what they would show in a novel way is that sequence effects can be obtained to the same extent as with S1-S2 sequences by using R1-S2 sequences (R1 being the prediction key-presses) when the R1 responses have fixed associations to the S2 stimuli. Such equivalence of responses and stimuli in evoking sequence effects might be explained, e.g., by Hommel et al.’s (2001) notion of *event files* composed of S-R associations, where already one element of the event file evokes the entire S-R complex.

Indeed, the present study cannot clearly distinguish between effects of conditional response frequencies and of stimulus sequences because the frequencies *f* and *i* (frequent and infrequent) of the match and mismatch responses to either S2 are directly determined by the frequencies *f* and *i* of the first events, such that S2-R2 combinations *F*→*match*, *I*→*mismatch*, *F*→*mismatch*, *I*→*match* imply the R1/S1-S2 sequences *f*→*F*, *f*→*I*, *i*→*F*, *i*→*I*. A convincing argument could be made if there were two conditions where the very same stimulus sequences require different responses. Then effects of response frequencies and stimulus sequences could be distinguished from each other. In this regard, a nearby manipulation would be to omit the overt key-press R2, the response to the target S2, because having no R2 is the more usual procedure in prediction tasks. We introduced R2, as an overt match/mismatch response, to make the task most similar to the S1-S2 comparison task. In an earlier study (Verleger and Śmigasiewicz, 2016) we had compared P3 amplitudes in prediction tasks between versions with and without R2. Amplitudes became somewhat smaller when the target events did not have to be confirmed by key-press than when external responses were required, by a constant amount across first-event and target frequencies. Yet, even then, these P3s were of appreciable size, like in all studies on the prediction task since Sutton et al. (1965) that did not require R2 responses. In contrast, when the same would be done with the comparison task, by not requiring responses to S2, we expect that S2-evoked P3 would largely disappear because S2 would lose its task relevance such that no P3b component would be evoked at all. This simple gedankenexperiment reminds us of the well-known evidence that, unlike Mismatch Negativity, P3 is governed by more factors than just stimulus sequence. Rather, the stimuli must be task-relevant (Johnson, 1986; see Ritter et al., 1999) which fact is captured by S-R link hypothesis in the notion that some S-R link must be reactivated upon perceiving S. Unfortunately, in order to avoid overloading our participants, we did not include such no-overt-response task versions in the present study.

### Larger P3 Amplitudes in the Prediction than the Comparison Task

Unexpectedly, S2-evoked P3 amplitudes were generally larger in the prediction than the comparison task. Since the two tasks were made as similar as possible to each other, this general difference cannot be ascribed to differences in response requirements to S2, in timing of R1/S1 and S2, or in payoff schemes. There were no consistent differences between tasks in pre-S2 negativity (CNV) that might have affected S2-evoked ERPs. Moreover, by their invariance across the different versions of the two tasks, these task differences cannot be ascribed to the mere presence or absence of S1 or R1.

However, as obvious from Figure 4, in contrast to the prediction task, P3 amplitudes were evoked in the comparison task by the S1 stimuli already, particularly by rare S1. Consequently, S2-evoked P3 might have decreased by habituation of P3 generators. However, these S1-evoked P3s were larger with rare than with frequent S1. Thus, if S2-evoked P3 was reduced in the comparison task by within-trial habituation, this habituation effect should be larger after rare than frequent S1, which was not the case. A remaining possibility is some overarching habituation in the comparison task due to the additional presence of the other P3 (S1-evoked) in a trial. Yet S1-evoked P3s were generally smaller in the *compare* task than in the *confirm and compare* task, therefore such overarching habituation should be less marked in this former task version. This was not the case.

Context updating hypothesis does offer an explanation for this effect, because this hypothesis assumes that the process reflected by target-evoked P3 serves for guiding future performance (Donchin, 1981; Donchin and Coles, 1988). This function may be required in the prediction task to arrive at a decision for the next trials about what stimulus should be predicted (see Munson et al., 1984, for an empirical test of this assumption) and not in the comparison task where the sequence of events cannot be controlled by participants. Yet, even here it is not clear why this effect is of same size for rare and frequent targets, because according to the hypothesis it is the rare stimuli above all that will lead to more updating of expectancies about the future.

A way to account for the generally larger P3 amplitudes in the prediction task may be in terms of motivational value of the target stimuli. Although we kept the payoff scheme equal between tasks, with matches yielding gains, it may make a subjective difference whether the match was achieved by one’s own key-press R1 (in the prediction task) or by the computer-generated S1 (in the matching task). Consequently, targets may be perceived as subjectively more relevant in the prediction task than in the matching task. Indeed, it has been shown that targets that arouse more motivation by being coupled to incentives may evoke larger P3 amplitudes (Begleiter et al., 1983; Baines et al., 2011; Flores et al., 2015). Thus, such an account is compatible with the literature. But we have to admit that, though not being in direct conflict with our S-R link hypothesis, this account does not have a direct relationship with this hypothesis either.

### S1-Evoked P3s

As just mentioned, P3s were evoked by rare S1s in the matching tasks, most distinctly in the *confirm and compare* version. In contrast, there was no S1-evoked P3 whatsoever in the *predict and see* task. This appears to be an interesting observation in its own right, reminding of the well-studied reduction of N1 components evoked by stimuli that were self-generated (e.g., Lange, 2011; Ford et al., 2014). Actually, it demonstrates the close connection of P3b to task relevance, in correspondence with several hypotheses. Here, we outline an account in terms of S-R link hypothesis. P3 was largest in the *confirm and compare* task because an immediate key-press response was required according to S1 identity in this task. When not being used in preceding trials, the link between a rare S1 and its response needs reactivation, reflected by P3. The smaller though still distinct P3s evoked by S1 in the pure* compare* task may have occurred because, even though no overt response was required to S1, S1 did specify the response required by S2 to be made with either the left or the right hand (with the finger then specified by S2). Again, the link from S1 to the hand less often used in preceding trials needs more reactivation, reflected by P3, with amplitudes being smaller than with direct responding because responses are not entirely specified and have to be delayed (Verleger et al., 2016b). Finally, in the *predict and see* task, links between R1 and S1 do not need any reactivation when perceiving S1 because the associated action R1, executed 30 ms before S1 appeared, was already fully active.

### Effects on RTs and Error Rates

Errors were committed almost exclusively with rare targets where participants pressed the *frequent* key instead. This tendency of simply perseverating with the frequent response, well known from go/no-go tasks (e.g., Perri et al., 2015; Nguyen et al., 2016) or choice-response tasks (Meckler et al., 2013; Verleger et al., 2016a) was seen both in the comparison and prediction tasks. The latter result is astonishing because one might think that participants would tend to press the key corresponding to the target they had predicted which would imply that less errors would be committed with *match* than *mismatch* responses. However, more errors were committed with infrequent S2s than frequent S2s even when infrequent S2s matched the prediction.

A major role of response frequency is also evident from the RT results: with the frequently used hand (used after having made a *frequent* prediction, or determined by the frequent S1) frequent (matching) responses were much faster than rare (mismatching) responses. Correspondingly, responses were slow with the rarely used hand (used after having made a *rare* prediction, or determined by the rare S1) both for the rare matching and the frequent mismatching responses.

Additionally, prediction outcome did have some effect on error rates, modulating the frequency effect. Most errors were committed with *I*→*mismatch* in the prediction task where, having predicted the frequent target, participants responded as if it was *F*→*match*, so it appears that prediction failure at least increased the error tendency induced by the frequent response. Additionally, the absence of this increase specifically in the *confirm S1* version of the comparison task means that this increase in the prediction task cannot be due to a priming effect of the preceding R1 response for making the frequent response to S2, because this would also occur in this version of the comparison task. Thus, this moderation of the frequency effect on errors seems due to prediction outcome. On the other hand, RTs were not affected by prediction outcome at all: RTs of mismatching responses were not slower in the prediction task than in the comparison task and, moreover, with infrequent S2, responses were faster to matches than to mismatches specifically in the comparison task but not in the prediction task.

To summarize, the role of expectancies reflected by predictions for affecting RTs and error rates seems only minor in comparison to the role of stimulus and response frequencies. This result provides further support for the prevailing role of stimulus and response frequencies, rather than of right or wrong outcome, for P3 in the prediction task.

## Conclusion and Limitations

In conclusion, by fitting the prediction task to a comparison-task framework, we provided an account within S-R link hypothesis for the occurrence of P3 components evoked by the apparently response-free targets. Of the two secondary findings, we could not give a convincing account for the obvious general difference in amplitudes between tasks, whereas the complete absence of P3 with rare S1 stimuli when triggered by rare key-press responses nicely fits S-R link hypothesis.

A limitation of this study is that, by design, there is a condition with very few trials and, accordingly, with a low signal/noise ratio. This is when the *infrequent* prediction was made and the infrequent stimulus was indeed presented (*I*→*match*). The *a priori* probability of this condition was 4% of 250 trials in each task version, i.e., 10 trials. By rejection of incorrectly responded and artifact-affected trials, this number could become even lower. Omitting this condition from analysis altogether would have compromised the ANOVA design where each factor had two levels and effects could, therefore, be easily interpreted. Although not introducing a systematic bias, low signal/noise ratio is bound to increase variability of the error term in ANOVAs such that effects might be prevented from becoming significant. In particular, it may be suspected that interactions of Task and Task Version with S2 Frequency and Conditional Response Frequency that now fell short of being significant at *p* = 0.05 (Table 3) might have become significant when signal/noise ratio would have been higher. Therefore, we repeated the ANOVA on P3 amplitudes, omitting the *I*→*match* condition. Lacking this fourth condition, we define *F*→*match*, *I*→*mismatch*, *F*→*mismatch* as three levels of an “event type” factor. Indeed, the interaction of Event Type × Task became significant, *F*_(2,30)_ = 3.9, ε = 0.81, *p* = 0.04, reflecting a difference between *F*→*match* and either other condition (*F*_(1,15)_ ≥ 5.1, *p* ≤ 0.04 in pair-wise comparisons): the difference between tasks (prediction > comparison) was smallest with *F*→*match*. Thus, it is doubtful whether the difference between tasks that we found so difficult to interpret is truly constant across the four S-R configurations.

Another limitation was the problem in comparing CNV amplitudes between tasks and task versions. It was hard to find a baseline interval common to all task versions. Usually, for measuring the pre-S2 level of CNV in an S1-S2 task, the interval before S1 would be taken as baseline. But S1 was earlier than in the other conditions in *confirm and compare* and there was no S1 at all in *predict*. The event 1s before S2 was R1 in three of the four tasks (*predict*, *predict and see*, *confirm and compare*) with R1 being accompanied by some positive-going potential, very distinct in *confirm and compare* as S1-evoked P3, but also visible in the two *predict* tasks. Again, like with the previous limitation, this is a principal problem of the design where we set out to compare R1-S2 tasks with S1-S2 tasks. Therefore, any statement about differences or similarities of CNV amplitudes between tasks must be taken with caution.

## Author Contributions

RV conceived the experiments, analyzed data, wrote the manuscript. SC and BS conducted the experiment. KS supervised the experiments and provided critical input to the manuscript. All authors have read and approved the submitted version.

## Conflict of Interest Statement

The authors declare that the research was conducted in the absence of any commercial or financial relationships that could be construed as a potential conflict of interest.

## Footnotes

^1^In Verleger and Śmigasiewicz (2016) we discussed the explanatory power of several concepts on stimulus attributes with regard to P3, and of several hypotheses on P3. The effects on P3 to be explained in that article were effects of task, of frequency, and of expectancy. The discussed concepts on stimulus attributes were whether stimuli are unexpected (Johnson, 1986), or awaited (Verleger, 1988), or primed (Kahneman and Tversky, 1982; Gonsalvez et al., 1999), or primed with their responses (Steiner et al., 2013; Verleger et al., 2014a), or relevant (Begleiter et al., 1983; Johnson, 1986). The discussed hypotheses were inhibition for focusing attention (Polich, 2007, 2012), memory storage (Polich, 2007, 2012), context updating (Donchin, 1981), closure/network reset (Desmedt and Debecker, 1979; Bouret and Sara, 2005), response facilitation (Nieuwenhuis et al., 2005), decision (O’Connell et al., 2012), reactivation of S-R links (Verleger et al., 2014a), and generating conscious representations (Dehaene et al., 2003).

^2^Therefore, for clarity, the 1.3 s interval in the pure *compare* task is split in Figure 1 into the 1 s intertrial interval, common to all tasks, and the following 0.3 s interval at trial onset. Figure 1 also makes clear that the setting of a 1.3 s interval in the *Confirm and Compare* task was by mistake. (1 s would have been correct). Fortunately, there were not any effects of this timing difference between task versions on the variables of interest.

^3^www.neurobs.com

^4^www.easycap.de

## References

[B1] Baines S. Ruz M. Rao A. Denison R. Nobre A. C. (2011). Modulation of neural activity by motivational and spatial biases. Neuropsychologia 49, 2489–2497. 10.1016/j.neuropsychologia.2011.04.029 21570417

[B2] Begleiter H. Porjesz B. Chou C. L. Aunon J. I. (1983). P3 and stimulus incentive value. Psychophysiology 20, 95–101. 10.1111/j.1469-8986.1983.tb00909.x 6828618

[B3] Bouret S. Sara S. J. (2005). Network reset: a simplified overarching theory of locus coeruleus noradrenaline function. Trends Neurosci. 28, 574–582. 10.1016/j.tins.2005.09.002 16165227

[B4] Dehaene S. Sergent C. Changeux J.-P. (2003). A neuronal network model linking subjective reports and objective physiological data during conscious perception. Proc. Natl. Acad. Sci. U S A 100, 8520–8525. 10.1073/pnas.1332574100 12829797 PMC166261

[B5] Desmedt J. E. Debecker J. (1979). Wave form and neural mechanism of the decision P350 elicited without pre-stimulus CNV or readiness potential in random sequences of near-threshold auditory clicks and finger stimuli. Electroencephalogr. Clin. Neurophysiol. 47, 648–670. 10.1016/0013-4694(79)90293-1 91495

[B6] Desmedt J. E. Debecker J. Manil J. (1965). Mise en évidence d’un signe électrique cérébral associé à la détection par le sujet d’un stimulus sensoriel tactile. Demonstration of an electric cerebral sign related to the subject’s detection of a sensory tactile stimulus. Bull. Acad. R. Méd. Belg. 5, 887–936. 5864251

[B7] Dien J. Spencer K. M. Donchin E. (2004). Parsing the late positive complex: mental chronometry and the ERP components that inhabit the neighborhood of the P300. Psychophysiology 41, 665–678. 10.1111/j.1469-8986.2004.00193.x 15318873

[B8] Donchin E. (1981). Surprise!…Surprise? Psychophysiology 18, 493–513. 10.1111/j.1469-8986.1981.tb01815.x 7280146

[B9] Donchin E. Coles M. G. H. (1988). Is the P300 component a manifestation of context updating? Behav. Brain Sci. 11, 357–374. 10.1017/s0140525x00058027

[B10] Duncan-Johnson C. C. Donchin E. (1977). On quantifying surprise: the variation of event-related potentials with subjective probability. Psychophysiology 14, 456–467. 10.1111/j.1469-8986.1977.tb01312.x 905483

[B11] Flores A. Münte T. F. Doñamayor N. (2015). Event-related EEG responses to anticipation and delivery of monetary and social reward. Biol. Psychol. 109, 10–19. 10.1016/j.biopsycho.2015.04.005 25910956

[B12] Ford J. M. Palzes V. A. Roach B. J. Mathalon D. H. (2014). Did I do that? Abnormal predictive processes in schizophrenia when button pressing to deliver a tone. Schizophr. Bull. 40, 804–812. 10.1093/schbul/sbt072 23754836 PMC4059422

[B13] Frith C. D. Done D. J. (1986). Routes to action in reaction time tasks. Psychol. Res. 48, 169–177. 10.1007/bf00309165 3823334

[B14] Gaeta H. Friedman D. Hunt G. (2003). Stimulus characteristics and task category dissociate the anterior and posterior aspects of novelty P3. Psychophysiology 40, 198–208. 10.1111/1469-8986.00022 12820861

[B15] Gonsalvez C. J. Gordon E. Grayson S. Barry R. J. Lazzaro I. Bahramali H. (1999). Is the target-to-target interval a critical determinant of P3 amplitude? Psychophysiology 36, 643–654. 10.1111/1469-8986.3650643 10442033

[B16] Hillyard S. A. Kutas M. (1983). Electrophysiology of cognitive processing. Annu. Rev. Psychol. 34, 33–61. 10.1146/annurev.ps.34.020183.000341 6338812

[B502] Hommel B. Müsseler J. Aschersleben G. Prinz W. (2001). The theory of event coding (TEC): a framework for perception and action planning. Behav. Brain Sci. 24, 849–937. 10.1017/S0140525X01000103 12239891

[B17] Johnson R. Jr. (1986). A triarchic model of P300 amplitude. Psychophysiology 23, 367–384. 10.1111/j.1469-8986.1986.tb00649.x 3774922

[B18] Kahneman D. Tversky A. (1982). Variants of uncertainty. Cognition 11, 143–157. 10.1016/0010-0277(82)90023-3 7198958

[B505] Kolossa A. Fingscheidt T. Wessel K. Kopp B. (2013). A model-based approach to trial-by-trial p300 amplitude fluctuations. Front. Hum. Neurosci. 6:359. 10.3389/fnhum.2012.00359 23404628 PMC3567611

[B19] Lange K. (2011). The reduced N1 to self-generated tones: an effect of temporal predictability? Psychophysiology 48, 1088–1095. 10.1111/j.1469-8986.2010.01174.x 21261634

[B20] Matt J. Leuthold H. Sommer W. (1992). Differential effects of voluntary expectancies on reaction times and event-related potentials: evidence for automatic and controlled expectancies. J. Exp. Psychol. Learn. Mem. Cogn. 18, 810–822. 10.1037//0278-7393.18.4.810 1385618

[B21] Meckler C. Carbonell L. Hasbroucq T. Burle B. Vidal F. (2013). To err or to guess: an ERP study on the source of errors. Psychophysiology 48, 415–422. 10.1111/psyp.12020 23445462

[B22] Munson R. Ruchkin D. S. Ritter W. Sutton S. Squires N. K. (1984). The relation of P3b to prior events and future behavior. Biol. Psychol. 19, 1–29. 10.1016/0301-0511(84)90007-3 6478001

[B23] Nguyen A. T. Moyle J. J. Fox A. M. (2016). N2 and P3 modulation during partial inhibition in a modified go/nogo task. Int. J. Psychophysiol. 107, 63–71. 10.1016/j.ijpsycho.2016.07.002 27394185

[B24] Nieuwenhuis S. Aston-Jones G. Cohen J. D. (2005). Decision making, the P3, and the locus coeruleus—norepinephrine system. Psychol. Bull. 131, 510–532. 10.1037/0033-2909.131.4.510 16060800

[B25] O’Connell R. G. Dockree P. M. Kelly S. P. (2012). A supramodal accumulation-to-bound signal that determines perceptual decisions in humans. Nat. Neurosci. 15, 1729–1735. 10.1038/nn.3248 23103963

[B508] Oldfield R. C. (1971). The assessment and analysis of handedness: the Edinburgh inventory. Neuropsychologia 9, 97–113. 10.1016/0028-3932(71)90067-4 5146491

[B26] Perri R. L. Berchicci M. Lucci G. Spinelli D. Di Russo F. (2015). Why do we make mistakes? Neurocognitive processes during the preparation-perception-action cycle and error detection. Neuroimage 113, 320–328. 10.1016/j.neuroimage.2015.03.040 25812715

[B27] Pfefferbaum A. Ford J. M. Weller B. J. Kopell B. S. (1985). ERPs to response production and inhibition. Electroencephalogr. Clin. Neurophysiol. 60, 423–434. 10.1016/0013-4694(85)91017-x 2580694

[B28] Polich J. (2007). Updating P300: an integrative theory of P3a and P3b. Clin. Neurophysiol. 118, 2128–2148. 10.1016/j.clinph.2007.04.019 17573239 PMC2715154

[B29] Polich J. (2012). “Neuropsychology of P300,” in The Oxford Handbook of Event-Related Potential Components, eds Luck S. J. Kappenman E. S. (New York, NY: Oxford University Press), 159–188.

[B30] Rac-Lubashevsky R. Kessler Y. (2016). Dissociating working memory updating and automatic updating: the reference-back paradigm. J. Exp. Psychol. Learn. Mem. Cogn. 42, 651–659. 10.1037/xlm0000219 26618910

[B31] Ritter W. Vaughan H. G. Jr. Costa L. D. (1968). Orienting and habituation to auditory stimuli: a study of short term changes in averaged evoked responses. Electroencephalogr. Clin. Neurophysiol. 25, 550–556. 10.1016/0013-4694(68)90234-4 4178749

[B510] Ritter W. Sussman E. Deacon D. Cowan N. Vaughan H. G. Jr. (1999). Two cognitive systems simultaneously prepared for opposite events. Psychophysiology 36, 835–838. 10.1111/1469-8986.3660835 10554596

[B32] Sawaki R. Katayama J. (2009). Difficulty of discrimination modulates attentional capture by regulating attentional focus. J. Cogn. Neurosci. 21, 359–371. 10.1162/jocn.2008.21022 18510441

[B33] Sommer W. Leuthold H. Matt J. (1998). The expectancies that govern the P300 amplitude are mostly automatic and unconscious. Behav. Brain Sci. 21, 149–154. 10.1017/s0140525x98210958

[B500] Sommer W. Leuthold H. Soetens E. (1999). Covert signs of expectancy in serial reaction time tasks revealed by event-related potentials. Percept. Psychophys. 61, 342–353. 10.3758/BF03206892 10089765

[B34] Squires N. K. Squires K. C. Hillyard S. A. (1975). Two varieties of long-latency positive waves evoked by unpredictable auditory stimuli in man. Electroencephalogr. Clin. Neurophysiol. 38, 387–401. 10.1016/0013-4694(75)90263-1 46819

[B501] Squires K. C. Wickens C. Squires N. K. Donchin E. (1976). The effect of stimulus sequence on the waveform of the cortical event-related potential. Science 193, 1142–1146. 10.1126/science.959831 959831

[B35] Steiner G. Z. Brennan M. L. Gonsalvez C. J. Barry R. J. (2013). Comparing P300 modulations: target-to-target interval versus infrequent nontarget-to-nontarget interval in a three-stimulus task. Psychophysiology 50, 187–194. 10.1111/j.1469-8986.2012.01491.x 23153378

[B36] Sutton S. Braren M. Zubin J. John E. R. (1965). Evoked-potential correlates of stimulus uncertainty. Science 150, 1187–1188. 10.1126/science.150.3700.1187 5852977

[B37] Tueting P. Sutton S. Zubin J. (1970). Quantitative evoked potential correlates of the probability of events. Psychophysiology 7, 385–394. 10.1111/j.1469-8986.1970.tb01763.x 5510812

[B38] Twomey D. M. Kelly S. P. O’Connell R. G. O. (2016). Abstract and effector-selective decision signals exhibit qualitatively distinct dynamics before delayed perceptual reports. J. Neurosci. 36, 7346–7352. 10.1523/JNEUROSCI.4162-15.2016 27413146 PMC4945659

[B503] Verleger R. (1988). Event-related potentials and cognition: a critique of the context updating hypothesis and an alternative interpretation of P3. Behav. Brain Sci. 11, 343–356. 10.1017/S0140525X00058015

[B504] Verleger R. (1991). The instruction to refrain from blinking affects auditory P3 and N1 amplitudes. Electroencephalogr. Clin. Neurophysiol. 78, 240–251. 10.1016/0013-4694(91)90039-7 1707797

[B39] Verleger R. (1998). Towards an integration of P3 research with cognitive neuroscience. Behav. Brain Sci. 21, 150–152. 10.1017/S0140525X98220954

[B44] Verleger R. Asanowicz D. Werner L. Śmigasiewicz K. (2015). Biased odds for head or tail: outcome-evoked P3 depends on frequencies of guesses. Psychophysiology 52, 1048–1058. 10.1111/psyp.12440 25882775

[B40] Verleger R. Baur N. Metzner M. F. Śmigasiewicz K. (2014a). The hard oddball: effects of difficult response selection on stimulus-related P3 and on response-related negative potentials. Psychophysiology 51, 1089–1100. 10.1111/psyp.12262 24981371

[B41] Verleger R. Metzner M. F. Ouyang G. Śmigasiewicz K. Zhou C. (2014b). Testing the stimulus-to-response bridging function of the oddball-P3 by delayed response signals and residue iteration decomposition (RIDE). Neuroimage 100, 271–280. 10.1016/j.neuroimage.2014.06.036 24960419

[B42] Verleger R. Cohen R. (1978). Effects of certainty, modality shift and guess outcome on evoked potentials and reaction times in chronic schizophrenics. Psychol. Med. 8, 81–93. 10.1017/s0033291700006656 635071

[B43] Verleger R. Śmigasiewicz K. (2016). Do rare stimuli evoke large P3s by being unexpected? A comparison of the oddball effects between standard-oddball and prediction-oddball tasks. Adv. Cogn. Psychol. 12, 88–104. 10.5709/acp-0189-9 27512527 PMC4975594

[B45] Verleger R. Grauhan N. Śmigasiewicz K. (2016a). Is P3 a strategic or a tactical component? Relationships of P3 sub-components to response times in oddball tasks with go, no-go and choice responses. Neuroimage 143, 223–234. 10.1016/j.neuroimage.2016.08.049 27570107

[B46] Verleger R. Grauhan N. Śmigasiewicz K. (2016b). Effects of response delays and of unknown stimulus-response mappings on the oddball effect on P3. Psychophysiology 53, 1858–1869. 10.1111/psyp.12756 27593167

[B47] Walter W. G. Cooper R. Aldridge V. J. McCallum W. C. Winter A. L. (1964). Contingent negative variation: an electric sign of sensorimotor association and expectancy in the human brain. Nature 203, 380–384. 10.1038/203380a0 14197376

